# Giant Catalytic DNA Particles for Simple and Intuitive Detection of Pb^2+^

**DOI:** 10.1186/s11671-016-1462-1

**Published:** 2016-05-11

**Authors:** Jieun Kim, Jong Bum Lee

**Affiliations:** Department of Chemical Engineering, University of Seoul, 163 Seoulsiripdaero, Dongdaemun-gu, Seoul 130-743 South Korea

**Keywords:** DNAzyme, Heavy metal detection, Biosensor

## Abstract

DNAzymes have been extensively studied as biosensors because of their unique functionality of cleaving substrate in the presence of metal ion cofactors. However, there are only a few reports on visual detection using gold nanoparticles. Here, we synthesized the DNAzyme microparticle (DzMP) (~1 μm) via rolling circle amplification for detection of Pb^2+^ without the help of other materials. Then, the substrate strands were labeled with two different fluorophores (6-carboxyfluorescein and Cy5) to visualize the DzMPs and to monitor the separation of substrate strands. Because of their large size, the decline in the number of fluorescent particles in the presence of Pb^2+^ could be successfully demonstrated by a fluorescence microscopy, presenting a new platform for heavy metal detection.

**Electronic supplementary material:**

The online version of this article (doi:10.1186/s11671-016-1462-1) contains supplementary material, which is available to authorized users.

## Background

Nucleic acids have attracted a great attention due to their versatile properties. Especially, various DNA enzymes (termed DNAzyme) have recently been developed by the systematic evolution of ligands by exponential enrichment (SELEX) since Breaker and Joyce presented the in vitro selection of catalytic DNA in 1994 [1]. These DNAzymes have unique properties that are capable of catalyzing bioorganic chemical reactions. For example, DNAzymes catalyzing RNA cleavage [1–3], ligation [4, 5], or modification of nucleic acids [6, 7] have been reported. They have been applied in various fields such as biochemistry, medicine, and analytical chemistry.

Particularly, DNAzymes cleaving DNA or RNA substrates in the presence of specific metal cofactor such as Pb^2+^, Mg^2+^, Cu^2+^, and UO_2_^2+^ have been widely studied [1, 8–10]. Also, Na^+^ [11, 12] or lanthanide [13, 14] -dependent DNAzymes have been recently reported. Because the toxicity of heavy metals is well known, these DNAzymes have often been used as a sensor to detect heavy metals. To be specific, fluorescent techniques [15, 16], polymerase chain reaction [17], electrochemical approaches [18], and surface-enhanced Raman scattering (SERS) [19, 20] have been applied for DNAzyme-based sensors. Also, the colorimetric sensors using gold nanoparticles were introduced [21]. However, there have been few studies on visual detection techniques and most of the conventional colorimetric detection methods need the immobilization of DNAzyme on gold nanoparticles [21–23].

**Fig. 1:**
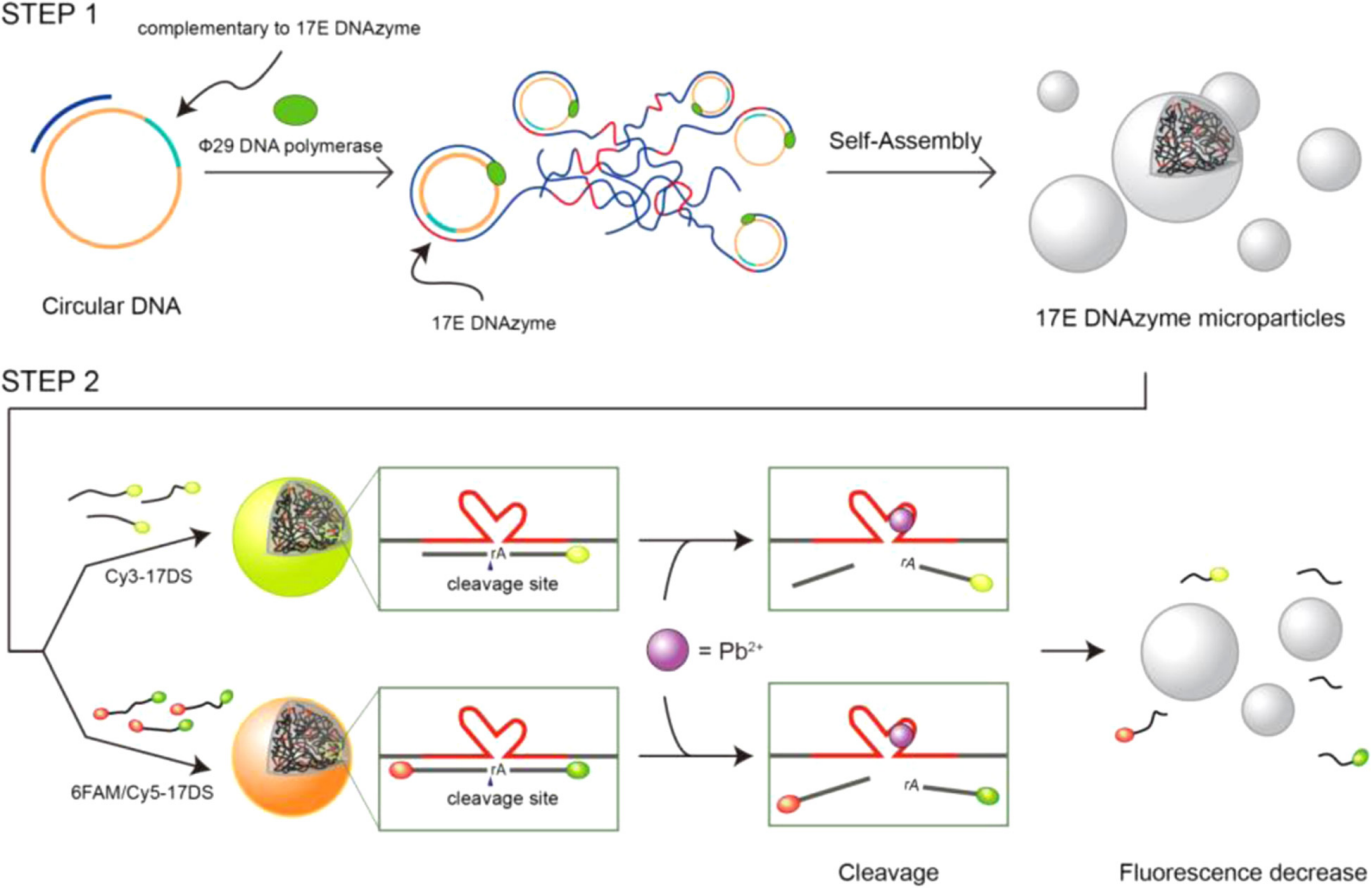
Schematic illustration of the construction of the 17DS/E DNAzyme microparticles and their catalytic activity. The DNA microparticles containing a large amount of 17E DNAzyme sites were synthesized via rolling circle amplification (RCA) (step 1). The 17DS substrate strands containing a cleavage site were added to the particle solution and then hybridized particles could be formed by denaturation and annealing. The substrate strands are cleaved when Pb^2+^ is added and the cleaved strands are separated from the particles (step 2)

Previously, the synthesis of DNA hydrogel via ligase-mediated reaction or rolling circle amplification (RCA) has been reported [24, 25]. Based on these enzymatic approaches, here we synthesized the giant catalytic DNA particles (~1 μm) without other inorganic core particles and successfully visualized the detection of Pb^2+^ utilizing a fluorescence microscope as illustrated schematically in Fig. 1. First, the DNAzyme microparticles (DzMPs) comprised of 17E DNAzyme for sensitive Pb^2+^ detection were produced by rolling circle amplification (RCA). Then, the fluorescently labeled 17DS substrate strands were hybridized with the generated particles. In the absence of Pb^2+^, the 17DS/E DzMPs emit bright fluorescence. However, in the presence of Pb^2+^, the particles turn off their light since the cleaved substrates are falling apart from the 17E DzMPs. This giant DNAzyme particle-based strategy enables detection of Pb^2+^ by fluorescence microscopy. In addition, DzMPs have numerous catalytic sites, and their sequences can be flexibly changed to have diverse functionalities. Therefore, this DzMP-based approach offers a simple detection method as well as a promise of a potent therapeutic tool for clinical applications.

## Methods

### Preparation of Circular DNA

All the oligonucleotides used in the study were purchased from the Integrated DNA Technologies (IDT, USA) (Table 1). To prepare the circular DNA, phosphorylated linear single-stranded DNA (ssDNA) that has complementary sequence to the 17E DNAzyme was designed and the primer ssDNA was designed to contain two complementary regions to each end of the linear ssDNA. For hybridization, equal concentration (3 μM) of the linear ssDNA and primer ssDNA were mixed in nuclease-free water. Next, the mixed solution was heated to 95 °C for 2 min and cooled gradually to 25 °C for 1 h. To connect the nick in the circular DNA, the solution was incubated overnight at room temperature with T4 DNA ligase (0.03 U/μL, Promega, USA) and ligase buffer (300 mM Tris-HCl (pH 7.8), 100 mM MgCl_2_, 100 mM dithiothreitol, and 10 mM adenosine triphosphate).

**Table 1:** DNA sequences for the fabrication of DNAzyme microparticles

Linear DNA	5′ - /Phosphate/CAA CTG TAG TGT GTT CAC GGT GCT GTA CTC ACT ATT TCG ACC GGC TCG GAG AAG AGA TGC ACT GAC AAG ACG TCA TAT CAA GTG TAT GGC AA - 3′	
Primer DNA	5′ - CAC TAC AGT TGT TGC CAT ACA C - 3′	
Dual-labeled 17DS substrate DNA	5′ - /6-FAM/ACT CAC TAT rA* GGA AGA GAT GCA CTG A/Cy5/ - 3′	*rA* represents the ribonucleotide adenosine
Cy3-labeled 17DS substrate DNA	5′- /Cy3/ACT CAC TAT rA* GGA AGA GAT G - 3′	*rA* represents the ribonucleotide adenosine

*rA represents the ribonucleotide adenosine

### Synthesis of 17E DNAzyme Microparticles

To synthesize the 17E DNAzyme microparticle (17E DzMP), the prepared circular DNA was mixed with Φ29 DNA polymerase (1 U/μL, Lucigen, USA), deoxyribonucleotide triphosphate (2 mM), and reaction buffer (40 mM Tris-HCl (pH 7.5), 50 mM KCl, 10 mM MgCl_2_, 5 mM (NH_4_)_2_SO_4_, and 4 mM dithiothreitol). For the RCA process, the mixed solution was incubated at 30 °C for 20 h. After brief sonication and washing several times using nuclease-free water, the 17E DzMP could be collected. Scanning electron microscopy (Hitachi, Japan, S-4200) was utilized to analyze the morphology of the DzMPs.

### Hybridization of 17DS Substrate with 17E DNAzyme Microparticles

To facilitate the confirmation of the hybridization of the 17E DzMP and 17DS substrate strands, the substrate was modified with the Cy3 fluorophore at the end of 5′ (Cy3-17DS). To determine the optimum ratio between 17E DzMP and Cy3-17DS, the different concentrations (200 nM, 1 μM, 5 μM each) of Cy3-labeled substrate strands was added into the 17E DzMP solutions. The denaturation and annealing processes were performed to produce Cy3-labeled 17DS/17E DNAzyme microparticles (Cy3-17DS/E DzMPs). The solution was heated to 95 °C for 2 min and cooled gradually to 25 °C for 1 h. The substrate residues were removed by washing several times. The fluorescence intensity of the 17DS/E DzMPs were measured using Nucleo Counter (Chemometec, Denmark, NC-3000). The fluorescent images of the particles were obtained by fluorescence microscopy (Nikon, Japan, Eclipse Ti).

### Gel Electrophoresis

To confirm the cleavage reaction, the various concentrations of Pb^2+^ solution (100 nM to 1 mM) was added to Cy3-17DS/E DzMP solutions. Then, to avoid the settling of DzMPs, the mixed solution was incubated using rotating oven at room temperature for 1 h. The catalytic activity of the DNAzyme was confirmed using DNA polyacrylamide gel electrophoresis (DNA-PAGE). Gel electrophoresis was carried out on a 10 % DNA-PAGE gel at 95 V at room temperature in Tris-borate-EDTA (TBE) buffer (0.89 M Tris-borate and 0.02 M EDTA, pH 8.3) for 40 min. First, the gel electrophoresis result was analyzed without DNA-specific dyes to monitor the Cy3-17DS substrate strands. Then, the resulting DNAzyme particles were analyzed after staining with GelRed (10^−4^ dilution of the stock solution, Biotium, USA).

### Detection of Pb^2+^ Using a Fluorescence Microscope

To observe DzMPs using a fluorescence microscope, dual-labeled substrate was used. The substrate strand which was labeled with a FAM fluorophore (6-carboxyfluorescein) at the 5′-end and a Cy5 at the 3′-end (6FAM/Cy5-17DS). To verify the optimal condition, different concentrations of 6FAM/Cy5-17DS (10 nM, 100 nM, 1 μM each) were mixed with 17E DzMPs and the same denaturation and annealing processes as above were performed. The fluorescence intensity of the 6FAM/Cy5-17DS/E DzMP was measured using Nucleo Counter (Chemometec). For the detection test, various concentrations of Pb^2+^ solution (100 nM to 1 mM) was added to the DzMP solution, and the mixed solution was incubated at room temperature using a rotating oven for 1 h. After cleavage reaction, the separation of the 6FAM/Cy5-17DS was monitored by utilizing fluorescence microscopy (Nikon).

## Results and Discussion

### Formation of Catalytic DNA Microparticles

**Fig. 2:**
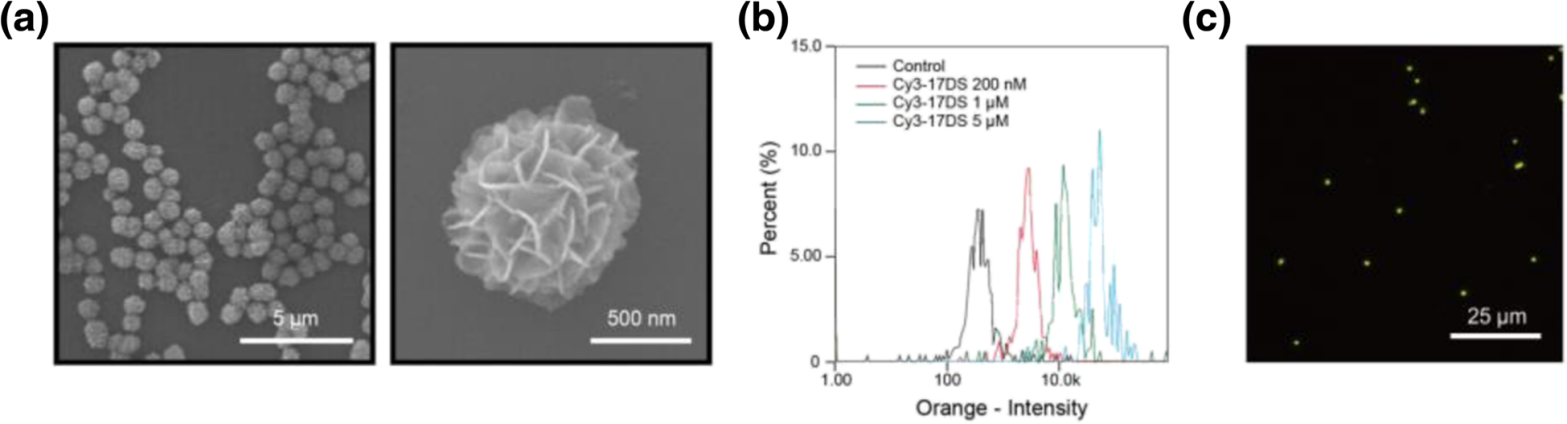
**a** SEM images of 17E DzMPs. **b** Histogram showing the fluorescence intensity of the Cy3-17DS/E DzMPs for various concentrations of the Cy3-17DSs. **c** Fluorescence microscopy image of the 17E-17DS particles which are hybridized with the 5 μM of Cy3-17DS

As illustrated in Fig. 1, the catalytic DNA particles were prepared from the designed circular template to generate 17E DNAzyme by RCA process. To observe the morphology and structure of the particles, scanning electron microscopy (SEM) was utilized. As seen in Fig. 2a, SEM image showed that the enzymatic particles have flower-like globular shape with uniform size (~1 μm). Because of the large surface area of the porous structure, numerous catalytic sites of the particles could participate in the enzymatic reaction. In addition, Cy3-labeled 17DS substrate strands (Cy3-17DS) were added to prepare 17E DzMPs to produce Cy3-17DS/E DzMPs. To determine the optimal ratio between substrates and DzMPs, three different concentrations (200 nM, 1 μM, and 5 μM each) of substrate solutions were tested. The hybridization between Cy3-17DS and 17E DzMPs was verified by utilizing image cytometry. As shown in Fig. 2b, fluorescence intensities of the particles increased as the concentration increased from 200 nM to 5 μM. Especially, there was a significant increase in intensity when the 5 μM of Cy3-17DS was used, compared to that of control 17E DzMPs. In addition, further study was performed using fluorescence microscopy, and the 17E particles which were mixed with 5 μM of Cy3-17DS showed bright fluorescence (Fig. 2c). These results indicated that the 17DS substrate strands were hybridized enough with the 17E particles which could catalyze the cleavage reaction. Also, the DzMPs still maintain their structure after hybridization with the 17DS substrate (see Additional file 1: Figure S1).

### Confirmation of Catalytic Activity of DzMPs

**Fig. 3:**
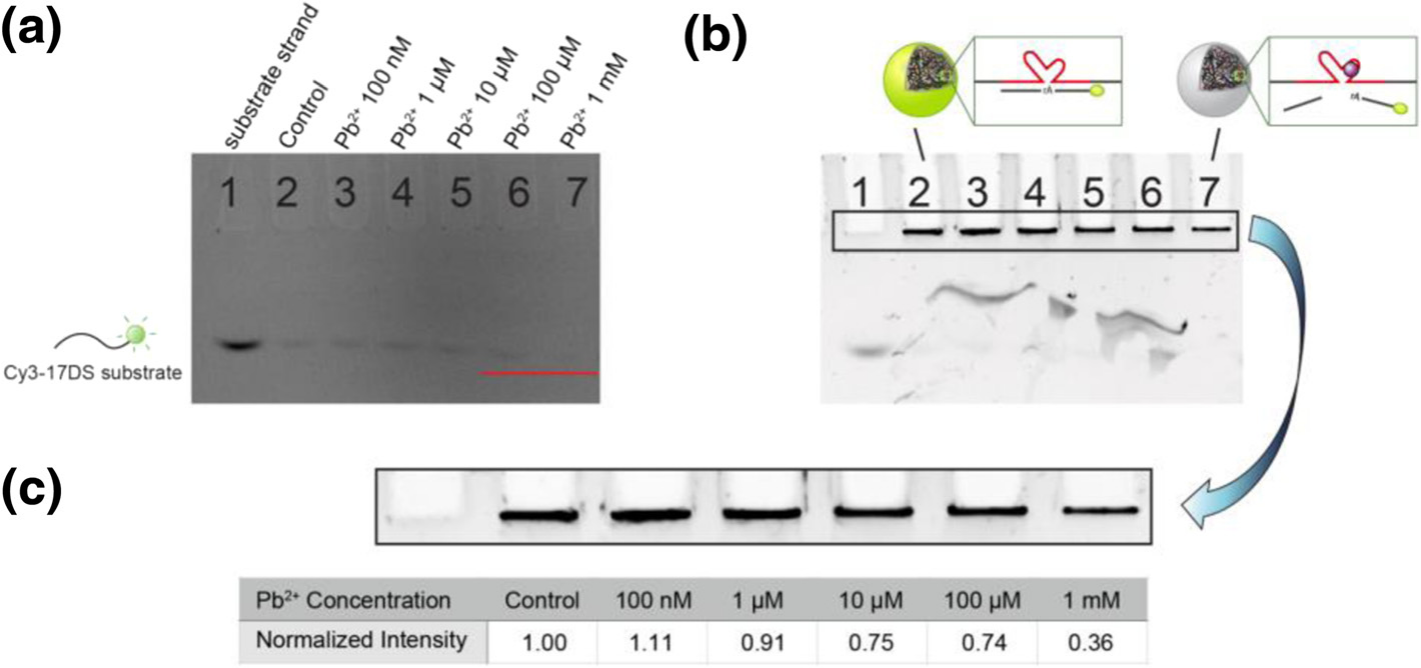
Gel electrophoresis results of the substrate strand (lane 1) and the 17DS/E DzMPs incubated with 100 nM (lane 3), 1 μM (lane 4), 10 μM (lane 5), 100 μM (lane 6), and 1 mM Pb^2+^ (lane 7) or untreated particles (lane 2). **a** The image that obtained without staining with the DNA-specific dyes. **b** The image that obtained after staining with the GelRed. **c** High magnification image of the highlighted area in **b** and the normalized fluorescence intensity of the particles

To confirm the catalytic activities and determine the sensitivity, various concentrations of Pb^2+^ (100 nM to 1 mM) were incubated with the Cy3-17DS/E DzMPs at room temperature for 1 h. After incubation, the solutions were analyzed by gel electrophoresis without purification to monitor the separated strands. Because of the presence of Cy3 fluorophore, the substrate strands could be analyzed without further staining with DNA-specific dyes. As seen in Fig. 3a, some 17DS substrates were detected at untreated control (lane 2). There are no significant changes at concentrations up to 100 μM. However, the substrate band was disappeared meaning cleavage of the substrate strands when the particles were treated with 1 mM Pb^2+^ (lane 7) and the cleaved substrate band was also observed (see Additional file 1: Figure S2). In addition, the previous gel was stained with GelRed to confirm the particles. Since the DzMPs have a size larger than the pore size, the particles were found within the wells (Fig. 3b). The intensities of the bands from lane 2 to lane 6 reveal little difference. On the other hand, in lane 7, the intensity of the band significantly decreased. This is probably due to the fact that the bulk of cleaved substrate strands were separated from the 17E DzMPs because of the catalytic activity of DNAzyme in the presence of Pb^2+^. For a quantitative study, the normalized intensities have shown in Fig. 3c. Under the concentration of 1 μM Pb^2+^, the differences between the intensities of untreated and Pb^2+^-treated samples were not substantial. However, the intensities were reduced up to about 25 % in the case of the concentration of 10 and 100 μM. In addition, the 1 mM treated particles showed an appreciable decrease of 64 % in the gel electrophoresis band intensity. Also, a gradual decrease in intensity of the DzMPs was demonstrated by additional experiment between 20 and 200 μM Pb^2+^ (see Additional file 1: Figure S3, Tables S1 and S2). These results suggest that the detection of Pb^2+^ was successfully achieved in the presence of Pb^2+^.

### Simple Detection of Pb^2+^ Using a Fluorescence Microscope

**Fig. 4:**
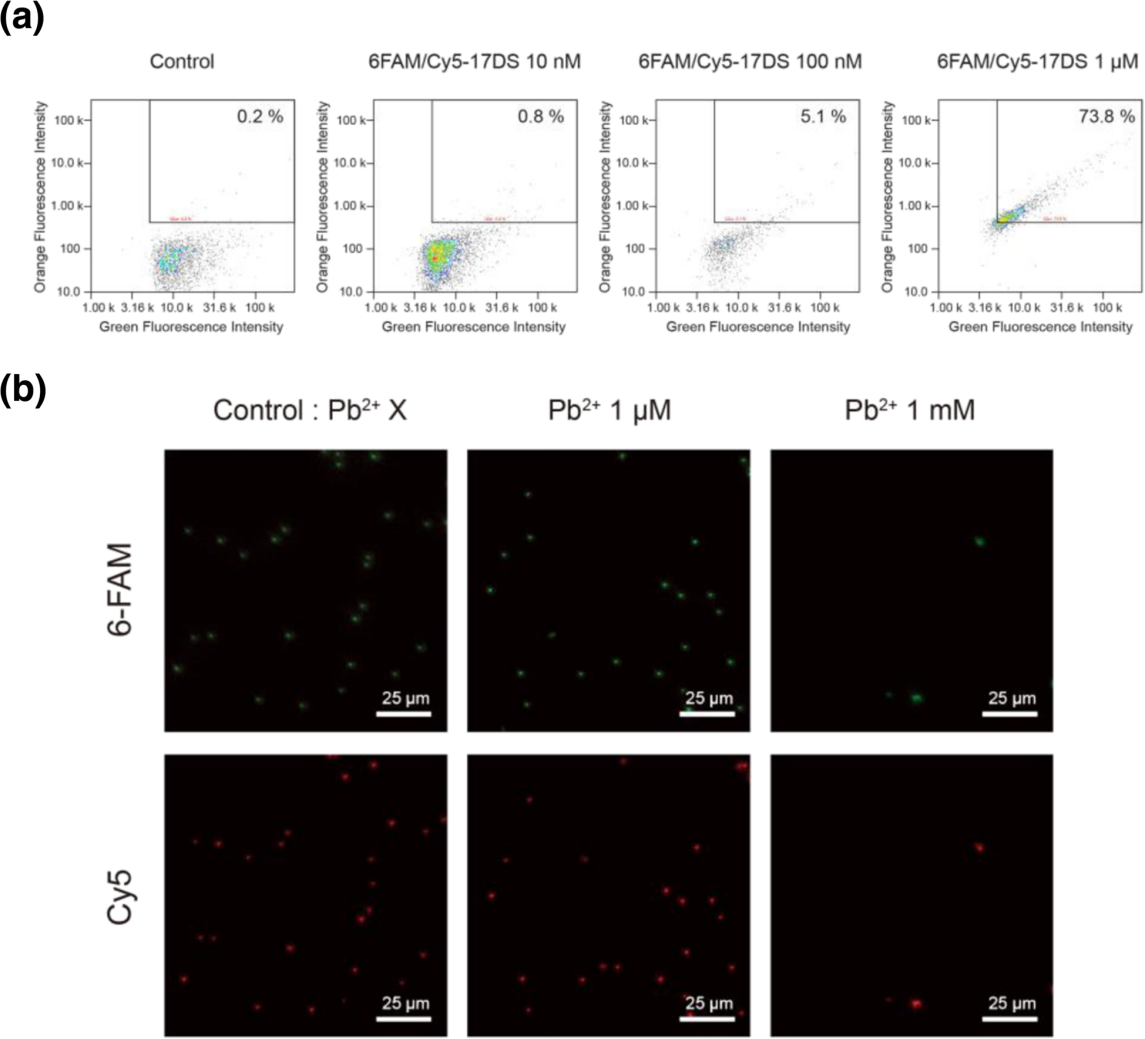
**a** Image acquisition cytometry plots of the 17E-17DS particles for the different concentrations of the 6FAM/Cy5-17DS substrates. **b** Fluorescence microscopy images of the 6FAM/Cy5-17DS/E DzMPs in the presence of different concentrations of Pb^2+^. The particles which are hybridized with the 1 μM of 6FAM/Cy5-17DS were incubated with lead ions at room temperature for 1 h

In order to develop a more convenient detection system, we designed the dual-labeled 17DS substrate. The substrate strand was labeled with a 6-carboxyfluorescein fluorophore (6-FAM) at the 5′-end and a Cy5 at the 3′-end (6FAM/Cy5-17DS). Therefore, more precise observations are available since the particles emit two different colors at once. To determine the proper concentration, different concentrations (10 nM, 100 nM, 1 μM) of 6FAM/Cy5-17DS were hybridized with the 17E DzMPs. The fluorescence intensities of the 6FAM/Cy5-17DS/E DzMPs were also measured by image cytometry. The results show that the number of the particles in the right upper quadrant was gradually increased as the concentration of 6FAM/Cy5-17DS increased (Fig. 4a). Especially, the scatterplot of 1 μM of substrate showed a clear linear form, and thus the DzMPs hybridized with 1 μM of 6FAM/Cy5-17DS (6FAM/Cy5-17DS/E DzMPs) were selected for the detection experiment. For the detection using a fluorescence microscope, 1 μM and 1 mM Pb^2+^ solution were investigated. As shown in Fig. 4b, it is confirmed that the untreated 6FAM/Cy5-17DS/E DzMPs emit both green and red fluorescence. There was not a particular change in the case of 1 μM Pb^2+^; however, when the 1 mM Pb^2+^ ions were treated, the number of fluorescent particles remarkably decreased. This means that the 6FAM/Cy5-17DS substrates were cleaved in the presence of Pb^2+^ and the cleaved substrates were separated from the DzMPs, leading to the reduction in fluorescence intensity. In addition, there were several fluorescent particles which have larger size than untreated control. It was probably caused by aggregation of particles with high concentration of positive charge ion. From these changes, a simple detection of lead ions could be achieved by the DzMP-based detection system.

## Conclusions

In conclusion, the DNAzyme microparticle having numerous DNAzyme sites was successfully synthesized. DNAzyme activity of the particles was confirmed by gel electrophoresis and quantification analysis and as low as 20 μM Pb^2+^ could be detected. Furthermore, dual-labeled substrate was designed for the simple detection platform. By utilizing the DNAzyme microparticles, the detection of lead ions was demonstrated based on the Pb^2+^-dependent DNAzyme chemistry. This strategy offers a simple and intuitive detection method with a detection limit of 1 mM Pb^2+^ and it could be applied in environmental applications [26, 27]. Although the sensitivity of this platform should be improved, the DNAzyme microparticles have a lot of advantages such as a large surface area, a high density of catalytic sites, and stability against nuclease [28]. Moreover, the functionality of DzMPs can be modified to contain diverse DNAzymes against a target in cancer cells by controlling sequences. Therefore, this catalytic DNA microparticles may well be useful not only as a detector but as a therapeutic medicine.

## Additional file

Additional file 1:
**Figure S1.** SEM images of 17DS/E DzMPs (left) and high magnification image (right). The 17E DzMPs were annealed with the 5 μM of Cy3-17DS. **Figure S2.** gel image showing cleavage activity of DNAzyme microparticles with different concentrations of Pb^2+^ after 1 h reaction. Lanes 1 and 2 indicate 17DS substrate strand and DzMPs without Pb^2+^ treatment. Lanes 3–7 correspond to 100 nM, 1 μM, 10 μM, 100 μM, and 1 mM Pb^2+^, respectively. Gel electrophoresis was carried out on a 3 % agarose gel at 95 V at 4 °C in 1× TAE buffer (40 mM Tris, 1 mM EDTA, 40 mM acetic acid) for 80 min. The gel image was obtained without DNA-specific dyes. **Figure S3.** fluorescence intensities of the Pb^2+^-treated DzMPs. Note: the slopes of the fitted linear plots were given as 1. **Table S1.** the slope and intercept of the linear fitting graphs. **Table S2.** the slope and intercept of the linear fitting graphs. (PDF 1278 kb)
