# Erratum to: Giant Catalytic DNA Particles for Simple and Intuitive Detection of Pb2+

**DOI:** 10.1186/s11671-016-1667-3

**Published:** 2016-10-10

**Authors:** Jieun Kim, Jong Bum Lee

**Affiliations:** Department of Chemical Engineering, University of Seoul, 163 Seoulsiripdaero, Dongdaemun-gu, Seoul 130-743 South Korea

## Erratum

Unfortunately, the original version of this article [1] did not contain the following acknowledgement:

‘This research was supported by the Global Innovative Research Center (GiRC) Program through the National Research Foundation of Korea (NRF) funded by the Ministry of Science, ICT & Future Planning (NRF-2012K1A1A2A01056093).”
