# Supplementary material for: Giant Catalytic DNA Particles for Simple and Intuitive Detection of Pb2+
Source: Nanoscale Res Lett. 2016 May 11;11:244. doi: 10.1186/s11671-016-1462-1 (PMC4864767; doi:10.1186/s11671-016-1462-1)
Supplement: Additional file 1: — Figure S1. SEM images of 17DS/E DzMPs (left) and high magnification image (right). The 17E DzMPs were annealed with the 5 μM of Cy3-17DS. Figure S2. gel image showing cleavage activity of DNAzyme microparticles with different concentrations of Pb2+ after 1 h reaction. Lanes 1 and 2 indicate 17DS substrate strand and DzMPs without Pb2+ treatment. Lanes 3–7 correspond to 100 nM, 1 μM, 10 μM, 100 μM, and 1 mM Pb2+, respectively. Gel electrophoresis was carried out on a 3 % agarose gel at 95 V at 4 °C in 1× TAE buffer (40 mM Tris, 1 mM EDTA, 40 mM acetic acid) for 80 min. The gel image was obtained without DNA-specific dyes. Figure S3. fluorescence intensities of the Pb2+-treated DzMPs. Note: the slopes of the fitted linear plots were given as 1. Table S1. the slope and intercept of the linear fitting graphs. Table S2. the slope and intercept of the linear fitting graphs. (PDF 1278 kb) [file 11671_2016_1462_MOESM1_ESM.pdf]

Supplementary Information

Giant Catalytic DNA Particles for Simple and Intuitive Detection of Pb2+

Jieun Kim [1] and Jong Bum Lee [1,*]

[1] Department of Chemical Engineering, University of Seoul, Seoul 130-743, South Korea;

* Author to whom correspondence should be addressed; E-Mail: jblee@uos.ac.kr;

Morphology of 17DS/E DzMPs

17DS/E DzMPs

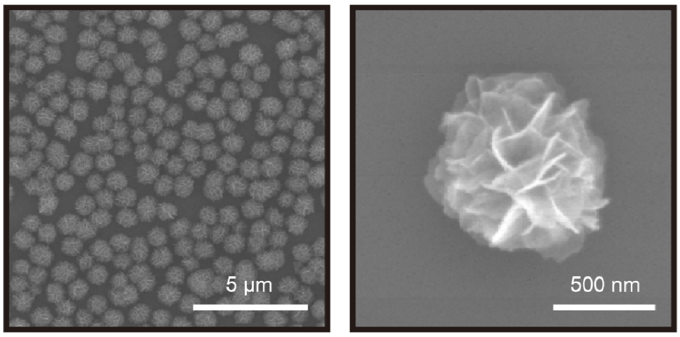

Figure S1. SEM images of 17DS/E DzMPs (left) and high magnification image (right). The 17E DzMPs were annealed with the 5 μM of Cy3-17DS.

Confirmation of DNAzyme activity

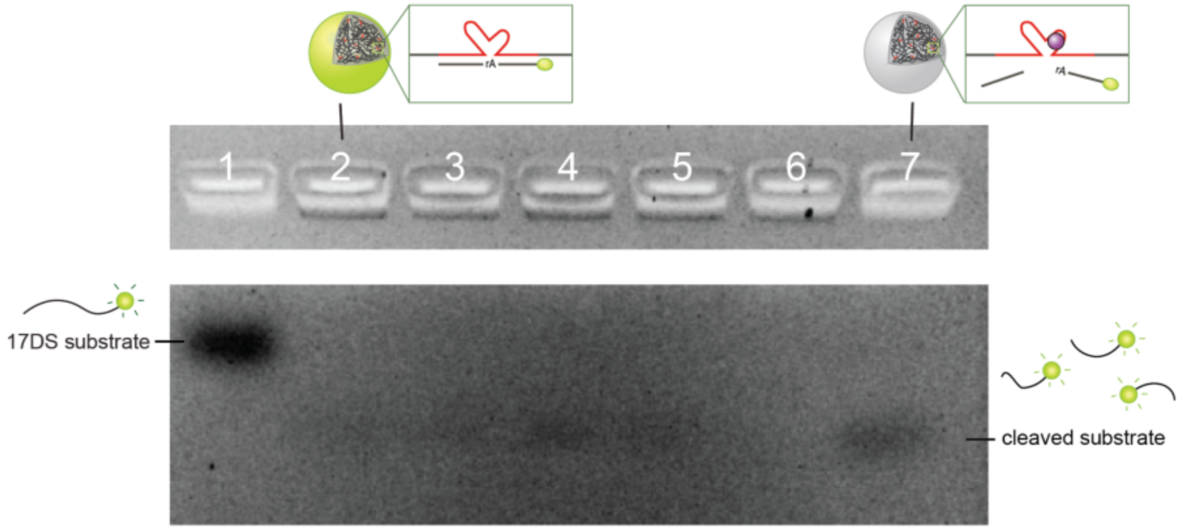

Figure S2. Gel image showing cleavage activity of DNAzyme microparticles with different concentrations of Pb^{2+} after 1 h reaction. Lane 1 and 2 indicate 17DS substrate strand and DzMPs without Pb^{2+} treatment. Lanes 3–7 correspond to 100 nM, 1 µM, 10 µM, 100 µM and 1 mM Pb^{2+} respectively. Gel electrophoresis was carried out on a 3% agarose gel at 95 V at 4℃ in 1x TAE buffer (40 mM Tris, 1mM EDTA, 40mM acetic acid) for 80 min. The gel image was obtained without DNA specific dyes.

Quantification analysis

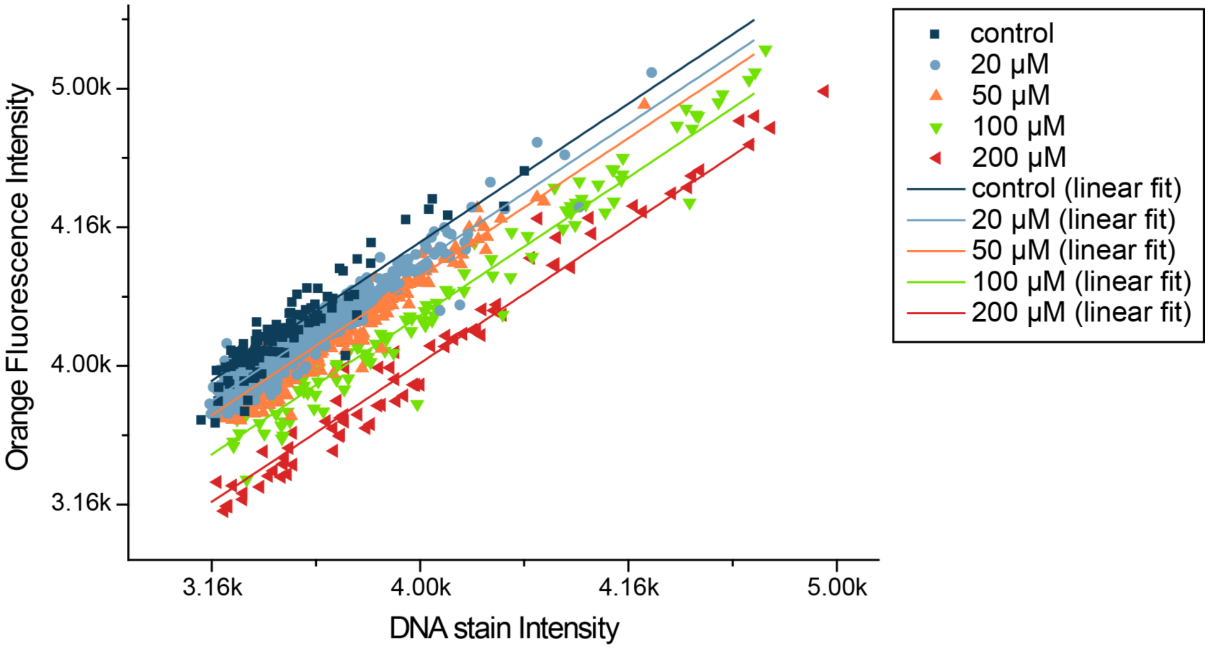

Figure S3. Fluorescence intensities of the Pb^{2+} treated DzMPs. Note: The slopes of the fitted linear plots were given as 1.

First, the fluorescence intensities of DzMPs were measured with Nucleo Counter (Chemometec, Denmark, NC-3000) and plotted with a logarithmic scale for the x-axis and y-axis. Then, the data were analyzed using linear regression analysis. The simple linear regression model is:

$$\log[Orange\ Intensity] = a \log[DNA\ stain\ Intensity] + b$$

A simple linear regression analysis reveals the following:

Table S1. The slope and intercept of the linear fitting graphs.

Pb^{2+} concentration	Slope [a]	Intercept [b]	Adj. R-square
0 μM	1.0934 ± 0.0458	0.1024 ± 0.1689	0.8465
20 μM	1.0124 ± 0.0106	0.3276 ± 0.0393	0.9201
50 μM	0.9928 ± 0.0124	0.3488 ± 0.0462	0.9115
100 μM	1.0509 ± 0.0195	-0.0250 ± 0.0794	0.9662
200 μM	1.0705 ± 0.0195	-0.2746 ± 0.0788	0.9767

As shown in table S1, every slope of fitted linear plots is about 1. Therefore, we reanalyzed the data with the following simple linear regression model to quantify the changes in orange fluorescence intensity.

$$\log[Orange\ Intensity] = \log[DNA\ stain\ Intensity] + b$$

And a simple linear regression analysis reveals the following:

Table S2. The slope and intercept of the linear fitting graphs.

Pb^{2+} concentration	Slope	Intercept [b]	Adj. R-square
0 μM	1	0.4463 ± 0.0064	0.8418
20 μM	1	0.3732 ± 0.0014	0.9201
50 μM	1	0.3222 ± 0.0017	0.9116
100 μM	1	0.1807 ± 0.0071	0.9643
200 μM	1	0.0093 ± 0.0078	0.9728

As shown in table S2, intercept of the linear fitting graphs were shifted downward as the concentration of Pb^{2+} increases. This result shows that the intensity of the DzMPs decreased because of the DNAzyme activity, and thus Pb^{2+} could be detected.
